# Editorial: Molecular biomarkers and imaging markers in the prediction, diagnosis, and prognosis of bladder cancer

**DOI:** 10.3389/fcell.2022.1014565

**Published:** 2022-10-14

**Authors:** Yongwen Luo, Fang-Ming Deng, Yi Zhang, Yu Xiao

**Affiliations:** ^1^ Department of Urology, Zhongnan Hospital of Wuhan University, Wuhan, China; ^2^ Department of Biological Repositories, Zhongnan Hospital of Wuhan University, Wuhan, China; ^3^ Human Genetics Resource Preservation Center of Hubei Province, Wuhan, China; ^4^ Department of Pathology, New York University Medical Center, New York, NY, United States; ^5^ Center of Life Sciences, Peking University, Beijing, China; ^6^ Euler Technology, Beijing, China

**Keywords:** bladder cancer, immunotherapy, metabolic reprogramming, immune microenvironment, biomarker

Bladder cancer (BLCA) is a common malignant tumor in the urinary system. BLCA is divided into muscle-invasive bladder cancer (MIBC) and non-muscle-invasive bladder cancer (NMIBC) (Sanli et al., 2017). Approximately 70% of BLCA patients are initially diagnosed with NMIBC, but 50%–70% of patients relapse after treatment, and 10%–20% of patients progress to MIBC. The 5-year survival rate for patients with MIBC is about 50% (Lenis et al., 2020; Wilson et al., 2022). Nowadays there is still a lack of public recognized, universally applicable diagnosis and prognostic markers for BLCA.

In this Research Topic, we compiled seventeen research articles and one review that summarized new advances about molecular biomarkers and imaging markers in the prediction, diagnosis, and prognosis of BLCA.

For the diagnosis of BLCA, Jeong and Ku systematically reviewed research progress of non-invasive diagnosis of BLCA. Urine cytology shows high sensitivity and specificity in high-grade urothelial cancer diagnosis. Moreover, nuclear matrix protein-22 (NMP-22), bladder tumor antigen (BTA), BTAstat and BTA-TRAK, UroVysion in Fluorescence *in situ* Hybridization, urine miRNA and urine cell-free DNA show high clinical application value for the diagnosis of BLCA. Xu et al. developed a novel urine cytology test (UCT) by mixing urine with mNPs (Nano-cytology) to harvest more tumor cells during UCT procedures. The Nano-cytology assay had a significantly improved sensitivity compared with UCT for detecting BLCA patients. It represents a promising tool for diagnosis of BLCA in clinical practice. Ye et al. utilized non-negative matrix factorization (NMF) algorithm to construct a radiomics signature based on CT images, and the radiomics signature is a potential biomarker to predict BCG response and relapse free survival (RFS) after BCG treatment in patients with high-risk NMIBC.

Increasing evidence have demonstrated that tumor microenvironment (TME), such as immune cells and cancer-associated fibroblasts (CAFs) affect tumor progression, prognosis and chemotherapy resistance (Biffi and Tuveson, 2019; Huang et al., 2022). Huang et al. identified three distinct immune cell infiltration (ICI) subtypes based on the TME immune infiltration pattern of 584 BLCA patients. The ICI score represented an effective prognostic predictor for evaluating the response to immunotherapy. Yang et al. identified four subtypes of BLCA based on immune profiling including immune ignorant, cold tumor, immune inactive, and hot tumor. CCL4 may be the key molecule functioning in immune cell infiltration in the hot tumor subtype. Moreover, neutrophils may function as an important suppressor in the TME of the immune ignorant and immune inactive subtypes. Chu et al. identified three types of TME patterns (stromal-activation subtype, immune-enriched subtype and immune-suppressive subtype). Then the tumor microenvironment signature (TMSig) was constructed by modified Lasso penalized regression. Patients in low-TMSig score groups had a better prognosis, higher M1 macrophage infiltration, better response to immunotherapy, and more similar molecular characteristics to the luminal (differentiated) subtype. Zheng et al. found that CD3E and LCK were potential biomarkers for MIBC. High-LCK and high-CD3E expression patients had a higher percentage of responders than the low-expression groups for immunotherapy. Tumor necrosis factor (TNF) family members play vital roles in cancer development and antitumor immune responses (Freeman et al., 2021). Li et al. developed and validated a robust TNF-based risk score, which could predict prognostic outcomes, TME, and molecular subtypes of BLCA. Ye et al. systematically assessed the DNA methylation modes in BLCA, and identified three DNA methylation modes. These modes are related to diverse clinical outcomes, immunophenotypes, aggressiveness, and immune responses of BLCA. DMRscore could serve as a signature to predict prognosis outcomes and immune responses. Taken together, these studies provided novel immunotherapy biomarkers and therapeutic targets for BLCA.

Additionally, amounting evidence indicates that ferroptosis may serve as a new target for BLCA (Kong et al., 2021; Lei et al., 2022). Xia et al. comprehensively evaluated the ferroptosis patterns of BLCA. They identified four distinct ferroptosis patterns, and verified ferroptosis is associated with TMB, TME immune cell infiltration, chemotherapy, and immunotherapy in BLCA. Wang et al. identified a prediction model containing five ferroptosis-related lncRNAs through integrated bioinformatics. This prediction model performed a good predictive ability, and can be used as an independent prognostic indicator.

Metabolic reprogramming is a unique hallmark of tumor cells. Accumulating evidence suggests that tumor metabolism plays a critical role in maintaining tumorigenesis and progression (Martínez-Reyes and Chandel, 2021; Raggi et al., 2022). Zhang et al. evaluated correlation between the metabolic status and the outcome of patients with BLCA using data from TCGA and GEO databases. Two clusters were identified using a consensus clustering algorithm based on an energy metabolism-related signature. The established energy metabolism-related gene signature was able to predict survival in patients with BLCA. Song et al. found iron metabolism is a pivot of tumor occurrence, progression, and TME in BLCA. They clustered the TCGA-BLCA cohort into four distinct iron metabolism patterns based on 95 prognosis-related iron metabolism-related genes (IMRGs), and then constructed the IMRG prognosis signature (IMRGscore), which could be utilized as an independent prognostic indicator.

Moreover, some authors have explored and studied biomarkers about progression, prognosis and treatment of BLCA. Tao et al. found that BRCC3 is upregulated in BLCA and indicates a negative survival prognosis. In BLCA cells, BRCC3 depletion dramatically attenuated cell proliferation, viability and migration. Mechanistically, BRCC3 binds with TRAF2 to activate NF-κB pathway. This finding points to BRCC3 as a potential target in BLCA patients. Mao et al. found ID2 expression was significantly downregulated in TCGA database and clinical samples, and high ID2 expression was associated with low-grade tumor staging and correlated with better overall survival, disease specific survival (DSS) and progress free interval (PFI). Mechanistically, ID2 acts as a tumor suppressor through PI3K/AKT signaling pathway to inhibit the progression and metastasis of BLCA. Du et al. found cancer-associated myofibroblasts (myCAFs) participate in extracellular matrix remodeling, tumor metabolism, cancer stemness, and oncological mutations. myCAFs have potential as potential diagnostic biomarkers and therapeutic targets for BLCA. Gu and Liang constructed a 15-top-prognostic gene-based signature based on TCGA-BLCA and GSE13507 cohorts, and this gene signature indicated a highly prognostic efficacy for BLCA. Moreover, the prognostic signature has a favorable predictive value for treatment with gemcitabine, doxorubicin, cisplatin, paclitaxel, and vinblastine. Song et al. identified key biomarkers in gemcitabine (GEM)-resistant BLCA and investigate their associations with tumor-infiltrating immune cells in a tumor immune microenvironment through integrative bioinformatics analysis. They reported that CAV1, COL6A2, FABP4, FBLN1, PCOLCE, and CSPG4 were critical biomarkers through regulating the immune cell infiltration in an immune microenvironment of GEM-resistance and could act as promising treatment targets for GEM-resistant MIBC.

In conclusion, the collections of research articles and reviews under this Research Topic present novel insights on the prediction, diagnosis, and prognosis of bladder cancer. It will hopefully encourage us to explore molecular targets from various perspectives and ultimately promote the diagnosis and treatment of BLCA.
